# Cardiometabolic outcomes in children and adolescents with West syndrome

**DOI:** 10.1186/s12887-021-02871-1

**Published:** 2021-09-18

**Authors:** Inbar Gilboa, Galit Israeli, Avivit Brener, Michal Yackobovitch-Gavan, Uri Kramer, Shimrit Uliel-Sibony, Yael Lebenthal

**Affiliations:** 1 Pediatric Endocrinology and Diabetes Unit, Dana-Dwek Children’s Hospital, Tel Aviv Sourasky Medical Center, 6423906 Tel Aviv, Israel; 2 Sackler Faculty of Medicine, Tel Aviv University, Tel Aviv, Israel; 3 Department of Epidemiology and Preventive Medicine, School of Public Health, Sackler Faculty of Medicine, Tel Aviv University, Tel Aviv, Israel; 4 Pediatric Neurology Institute, Dana-Dwek Children’s Hospital, Tel Aviv Sourasky Medical Center, Tel Aviv, Israel

**Keywords:** Adrenocorticotropic hormone (ACTH), Dyslipidemia, Hypertension, Infantile spasms, Metabolic outcomes, Obesity

## Abstract

**Background:**

West syndrome is a convulsive disorder of infancy with unique seizures and a characteristic background electroencephalograph pattern. Adrenocorticotropic hormone (ACTH) is effective in spasm cessation, yet metabolic consequences of this therapeutic agent in childhood have not been published.

**Methods:**

In this observational study we explored the cardiometabolic outcomes of 117 children with West syndrome (78 ACTH-treated and 39 non-ACTH-treated) monitored at a single medical center from 1995 to 2019 (median follow-up 7.2 years). Outcomes included the prevalence of cardiometabolic derangements (obesity, hypertension, and dyslipidemia) during infancy (< 2 years), early childhood (2–6 years), and childhood/adolescence (6–18 years).

**Results:**

The rates of metabolic derangements during infancy in the West syndrome cohort were high compared to childhood/adolescence (obesity 27.3 % vs. 3.3 %, [*p* = 0.010], diastolic hypertension 48.8 % vs. 5.1 % [*p* < 0.001], hypertriglyceridemia 71 % vs. 40 % [*p* = 0.008], low high-density lipoprotein cholesterol [HDL-c] 54.2 % vs. 12.9 % [*p* = 0.001], and elevated triglycerides/HDL-c ratios 62.5 % vs. 12.9 % [*p* < 0.001]). The proportion of systolic and/or diastolic blood pressure levels categorized as hypertensive was 58.5 % during infancy, 48.1 % during early childhood, and 26.3 % during childhood/adolescence. ACTH-treated patients had higher weight and weight-to-length z-scores and higher triglyceride levels during infancy compared to non-ACTH-treated patients (*p* = 0.008, *p* = 0.001, and *p* = 0.037, respectively), and higher triglyceride levels during early childhood (*p* = 0.050), with no significant group differences during childhood/adolescence.

**Conclusions:**

Children with West syndrome apparently have an increased prevalence of cardiometabolic derangements more pronounced in infants and in ACTH-treated patients. These findings highlight the need to monitor these children for cardiometabolic derangements, even though these cardiometabolic abnormalities are transitory and tend to decrease with time. The health implications of cardiometabolic derangements during critical windows of growth and development warrant further investigation.

## Background

West syndrome is a convulsive disorder of infancy, that may result from various etiologies, characterized by the combination of epileptic spasms (infantile spasms) and a characteristic electroencephalograph (EEG) pattern (“hypsarrhythmia”), as well as subsequent or concurrent arrest of psychomotor development [1–5]. Seizure onset is usually within the first year of life, with a peak at age 3–5 months [1]. The incidence of West syndrome is 0.16–0.42 per 1000 live births [1]. The seizures are often refractory to treatment with most conventional antiepileptic drugs [1, 6].

In 2009, the U.S. Food and Drug Administration approved adrenocorticotropic hormone (ACTH) and vigabatrin as the first line of therapy for West syndrome, and in 2012 evidence-based updated practice guidelines indicated no clear preference of ACTH and vigabatrin [7], with the exception of children who had underlying tuberous sclerosis, for which vigabatrin was the reported treatment of choice [1, 5]. The proposed mechanisms of action of ACTH therapy in infantile spasms are the reduction of neuronal excitability by inducing steroid release and/or by direct steroid-independent action on melanocortin receptors [8], as well as suppression of corticotropin-releasing hormone, an excitant neuropeptide [4]. There is no precise therapeutic protocol for ACTH, but most of the evidence suggests 2 weeks of treatment followed by dose tapering [5, 9].

Hormonal treatment with ACTH and corticosteroids may result in acute to subacute marked metabolic changes, such as weight gain, inhibition of growth, elevated blood pressure (BP), and insulin resistance [10, 11]. To date, there have been no published reports on the long-term evolution of these metabolic derangements associated with this therapeutic agent in the treatment of West syndrome. The objectives of this study were to describe the cardiometabolic characteristics in patients with West syndrome from infancy to childhood/adolescence, and to determine whether they differ among patients treated with ACTH during infancy compared to those not treated with ACTH.

## Methods

This observational real-life study of children and adolescents with a history of West syndrome was based upon data collected from medical records of all patients treated in our Epilepsy Center from 1995 to 2019. The study protocol was approved by our Institutional Review Board, which waived informed consent of the participants since the data retrieved from the medical records were anonymous to the researchers. The data were handled in accordance with the principles of good clinical practice.

**Fig. 1:**
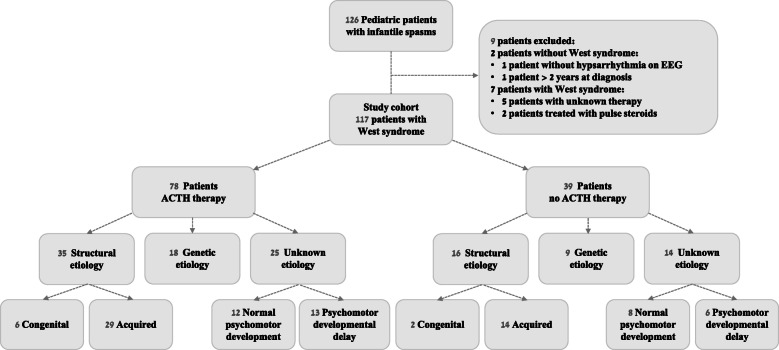
The study population of 117 patients with West syndrome classified according to treatment regimen (ACTH and no ACTH therapy) and the revised International League Against Epilepsy classification. No patients were classified as being in the “metabolic etiology” group. Unknown etiology is subcategorized as with or without psychomotor developmental delay

The study population was comprised of 117 patients with West syndrome classified according to treatment regimen (ACTH and no ACTH therapy) and the revised International League Against Epilepsy classification (structural, metabolic, genetic, or unknown) [12] (Fig. 1). Included in the study were children and adolescents diagnosed with West syndrome during infancy (aged 3–24 months) that was confirmed by epileptic spasms and a characteristic EEG pattern of hypsarrhythmia [1, 3]. Excluded from the study were 2 patients treated with pulse steroids in the non-ACTH group, and 5 patients whose data on the administered therapy were unclear.

In our center, ACTH is the first-line therapy in West syndrome, with the exception of cases of tuberous sclerosis and some selected cases of infants with a suspected structural, metabolic or genetic cause. The parents were made aware of the ACTH treatment protocol, the therapeutic course and the potential adverse effects. Vigabatrin was always the first-line therapy in patients with an underlying etiology of tuberous sclerosis. The therapeutic protocol for ACTH used at our institution was high-dose intramuscular tetracosactide (Synacthen Depot®, Novartis) 120 to 150 IU/m^2^/dose given on alternate days for 1–2 weeks followed by dose tapering. Treatment failure was defined when ACTH treatment failed after 2 weeks of the high-dose protocol.

Outcome measures included anthropometric parameters (weight, length/height, and weight-to-length/BMI z-scores), and prevalence of cardiometabolic derangements (obesity, hypertension, and dyslipidemia [hypertriglyceridemia, low levels of high-density lipoprotein cholesterol (HDL-c) and elevated triglyceride to HDL-c ratio]) at 3 time points: infancy (< 2 years), early childhood (2–6 years), and childhood/adolescence (6–18 years). The patient’s anthropometric and metabolic data during infancy was extracted at least 2 months following ACTH cessation.

The information in the medical files contained both parent-reported information and physicians’ notes on diagnoses, management, and surveillance. Paper-based medical files were used prior to 2000, after which all hospital medical records gradually became electronic, with additional access to the individual’s health maintenance organization (HMO) laboratory data since 2007. The retrieved information included sociodemographic characteristics (age at last hospital visit, sex, country of origin, socioeconomic position [SEP] by home address), and West syndrome-related characteristics (conception [spontaneous/assisted], prenatal and perinatal history (pregnancy [singleton/twin], delivery [vaginal/elective cesarean section/emergency cesarean section/vacuum extraction], APGAR score [1 and 5 min], gestational age [weeks], birth weight [grams], corrected birth weight z-score, head circumference [cm], head circumference z-score), age at diagnosis, etiology [structural/metabolic/genetic/unknown], cerebral palsy [yes/no], gross motor function classification system, brain imaging [magnetic resonance imaging (MRI)/computed tomography (CT)], psychomotor development [normal/mild delay/moderate delay/severe delay], and ACTH therapy and other medications). Psychomotor development was determined according to formal assessments by trained personnel from a child development institute, or by clinical evaluation of a pediatric neurologist. Cardiometabolic data included systolic and diastolic BP, anthropometric measurements (weight, length/height and calculated weight-to-length/body mass index [BMI]), and laboratory evaluation (glucose and lipid profile [total cholesterol, triglycerides (TG), high-density lipoprotein (HDL-c)], and thyroid function tests [thyroid-stimulating hormone (TSH) and free thyroxine (fT4)]). The TG:HDL-c ratio was calculated [13]. Details on medications and dietary interventions that could affect anthropometric and cardiometabolic outcomes were also collected.

Z-scores of anthropometric measurements were calculated with PediTools Electronic Growth Chart Calculators, based on CDC growth charts, and those scores were adjusted for gestational age in preterm infants [14]. Z-scores of anthropometric measurements in patients with cerebral palsy were calculated with cerebral palsy-specific growth charts [15]. Weight status was categorized as underweight for weight-to-length/BMI values < 5th percentile, normal for weight-to-length/BMI between 5th and 84th percentiles, overweight for weight-to-length/BMI between 85th and 94th percentiles, and obese for weight-to-length/BMI ≥ 95th percentile [16]. Corrected birth weight z-scores were calculated with PediTools Electronic Growth Chart Calculators based on the Fenton growth chart for preterm infants [14]. Appropriate for gestational age (AGA) birth weight was defined as corrected birth weight z-scores of -1.88 to 1.88, small for gestational age (SGA) as birth weight z-scores < -1.88, and the large for gestational age (LGA) as birth weight z-scores > 1.88. BP percentiles were calculated by means of an online age-based pediatric BP calculator [17]. BP was defined as normal when BP values were < 90th percentile, elevated when either systolic and/or diastolic BP levels were from the ≥ 90th to 95th percentile, and hypertensive when either the systolic and/or the diastolic BP level were ≥ 95th percentile [18]. Dyslipidemia was defined as a TG level ≥ 100 mg/dL, HDL-c ≤ 40 mg/dL or TG/HDL-c ≥ 3 [19, 20].

Ethnicity was defined as the birthplace of the parents or grandparents (if the parents were born in Israel) and categorized according to country or region. SEP was defined by home address according to the Israel Central Bureau of Statistics’ Characterization and Classification of Statistical Areas within Municipalities and Local Councils by the Socio-Economic Level of the Population 2015 [21]. The SEP by cluster of localities of residence ranged from 1 to 10; with 1 being the lowest rating and 10 the highest. The SEP index is an adjusted calculation of 14 variables that measure social and economic levels in the domains of demographics, education, standard of living, and employment (ranging from − 2.797 to 2.590).

### Statistical Analyses

The data were analyzed with Statistical Package for the Social Sciences software version 27 (SPSS Inc., Chicago, IL). All statistical tests were 2-sided. The Kolmogorov-Smirnov test and the Shapiro-Wilk test were applied to test the normality of continuous data. The data are expressed as means ± standard deviations (SDs) for normally distributed variables and median and interquartile range (IQR) for skewed distribution. Pearson’s chi-square test or Fisher’s exact test were performed to compare the distribution of categorical variables between the treatment groups (ACTH and non-ACTH) as appropriate. The independent sample t-test or independent sample Mann-Whitney were performed to compare between treatment groups for continuous variables with normal or skewed distribution, as appropriate. A 1-sample t-test was performed to test the null hypothesis that the participant’s mean z-score equals zero. Univariate logistic regression analysis was applied to determine odds ratio (OR) and 95 % confidence interval (CI) between ACTH therapy and cardiometabolic factors (BMI z-scores, systolic BP percentiles, diastolic BP percentiles, TG levels, HDL-c levels, and TG:HDL-c ratios). A *p* value of ≤ 0.05 was considered significant.

## Results

The data on sociodemographic characteristics, maternal conditions during pregnancy and perinatal characteristics of the entire West syndrome cohort (117 patients, 53 % males, mean age at diagnosis 6.4 ± 3.5 months) are presented in Table 1. The characteristics of the cohort stratified according to treatment group (ACTH-treated and non-ACTH-treated) are presented in Table 2. Sex, SEP and ethnic distribution did not differ between treatment groups. The distribution of residency differed between treatment groups: more ACTH-treated patients were Israeli residents compared to the non-ACTH-treated group (*p* = 0.043). The analyzed perinatal characteristics included maternal age, mode of conception, number of fetuses, mode of delivery, gestational age, adjusted birth weight, and birth weight categories, and they did not differ between treatment groups. Patients in the ACTH-treated group were characterized by higher rates of birth at term (*p* = 0.046) and larger adjusted head circumferences compared to those in the non-treated group albeit within the normal range (*p* = 0.005).

**Table 1:** Sociodemographic and perinatal characteristics of the West syndrome cohort

Number	117
Male sex, n (%)	62 (53.0)
Socioeconomic position*	*n* = 89
Cluster	6.0 ± 2.4
Index	0.435 ± 1.065
Ethnicity, n (%)	111
Former Union of Soviet Socialist Republics	31 (27.9)
North Africa	26 (23.4)
Europe	22 (19.8)
Mixed ethnicity	23 (20.7)
Israeli Arab	7 (6.3)
Other	2 (1.8)
Maternal age at offspring birth, years	29.8 ± 5.3
Maternal conditions during pregnancy, n (%)	*n* = 82
Healthy	73 (89.0)
Hypercoagulability	4 (4.9)
Gestational diabetes mellitus	3 (3.7)
Hypothyroidism	1 (1.2)
Inflammatory bowel disease	1 (1.2)
Mode of conception, n (%)	*n* = 83
Spontaneous	79 (95.2)
Assisted fertilization	4 (4.8)
Pregnancy, n (%)	*n* = 83
Twin	11 (13.3)
Intrauterine growth, n (%)	*n* = 54
Intrauterine growth retardation	4 (7.4)
Mode of delivery, n (%)	*n* = 74
Vaginal	44 (59.5)
Cesarean section, elective	15 (20.3)
Cesarean section, emergency	11 (14.9)
Vacuum extraction	4 (5.4)
Gestational age	*n* = 85
Gestational age, weeks	39 (36–40)
Preterm, < 37 weeks gestation, n (%)	25 (29.4)
Term, 38–42 weeks gestation, n (%)	54 (63.5)
Postterm, > 42 weeks gestation, n (%)	6 (7.1)
Newborn	*n* = 78
APGAR score, 1 min	9 (6–9)
APGAR score, 5 min	10 (8–10)
Birth parameters	*n* = 78
Birth weight, z-score	-0.33 ± 0.95
Small for gestational age, n (%)	5 (6.4)
Appropriate for gestational age, n (%)	72 (92.3)
Large for gestational age, n (%)	1 (1.3)
Head circumference, z-score	-0.59 ± 0.56

Data are expressed as number and (percent), mean ± standard deviation, median (interquartile range). Socioeconomic position (SEP) was determined by cluster of localities of residence, ranging from 1 to 10; with 1 being the lowest rating and 10 the highest. The SEP index is an adjusted calculation of 14 variables that measure social and economic levels in the domains of demographics, education, standard of living, and employment (ranging from the lowest − 2.797 to the highest 2.590). The ethnicity was defined as the birthplace of the parents or grandparents (if the parents were born in Israel) and categorized according to country or region. *Missing data: SEP (n = 28, tourist medicine from Russia and the Ukraine [n = 26] and Israeli residents with paper medical files [*n* = 2]). Abbreviations: *APGAR*, appearance pulse grimace activity respiration; *n* number

**Table 2:** Comparative analysis of the characteristics of 117 patients with West syndrome according to treatment group

	ACTH treated	non-ACTH treated	*P* value
Number	78	39	
Male sex, n (%)	42 (53.8)	20 (51.3)	0.799
Socioeconomic position	n = 64	n = 25	
Cluster	6.0 ± 2.4	5.9 ± 2.3	0.909
Index	0.447 ± 1.086	0.406 ± 1.031	0.871
Residency status, n (%)			
Israeli	65 (83.3)	26 (66.7)	**0.043**
Tourists	13 (16.7)	13 (33.3)	
Gestational age	n = 66	n = 19	
Gestational age, weeks	39 (36–40)	39.5 (28–41)	0.560
Preterm < 37 weeks’ gestation, n (%)	17 (25.8)	8 (42.1)	**0.050**
Term 38–42 weeks’ gestation, n (%)	46 (69.7)	8 (42.1)	
Postterm > 42 weeks gestation, n (%)	3 (4.5)	3 (15.8)	
Birth parameters			
Birth weight, z-score	-0.34 ± 0.94	-0.27 ± 1.02	0.787
Head circumference, z-score	-0.45 ± 0.47	-1.37 ± 0.40	**0.005**
West syndrome			
Age at diagnosis, months	5.9 ± 3.0	7.5 ± 4.2	**0.031**
Etiology, n (%)	n = 78	n = 39	
Unknown, normal development	12 (15.4)	8 (20.5)	0.991
Unknown, developmental delay	13 (16.7)	6 (15.4)	
Genetic syndrome	18 (23.1)	9 (23.1)	
Structural, congenital	6 (7.7)	2 (5.1)	
Structural, acquired	29 (37.2)	14 (35.9)	
Metabolic	0 (0)	0 (0)	
Brain imaging - MRI/CT, n (%)	n = 72	n = 31	
Normal	14 (19.4)	5 (16.1)	0.693
Pathologic	58 (80.6)	26 (83.9)	
Cerebral palsy, n (%)	26 (33.3)	13 (33.3)	1
Psychomotor development, n (%)	n = 69	n = 29	
Normal	10 (14.5)	0 (0)	**0.040**
Mild developmental delay	9 (13)	2 (6.9)	
Moderate developmental delay	21 (30.4)	16 (55.2)	
Severe developmental delay	29 (42)	11 (37.9)	

Data are expressed as number and (percent), mean ± standard deviation, median (interquartile range). Socioeconomic position (SEP) was determined by cluster of localities of residence, ranging from 1 to 10; with 1 being the lowest rating and 10 the highest. The SEP index is an adjusted calculation of 14 variables that measure social and economic levels in the domains of demographics, education, standard of living, and employment (ranging from the lowest − 2.797 to the highest 2.590). *P* values are between the ACTH- and non-ACTH-treated groups. Bold indicates significant

*Maternal age at delivery was available for 41 mothers of infants in the ACTH-treated group and for 8 mothers in the non-ACTH treated group. Abbreviations: *ACTH* adrenocorticotropic hormone; *CT* computed tomography; *MRI* magnetic resonance imaging; *n* number

The West syndrome-related characteristics of our patients stratified by treatment group revealed that, on average, children in the ACTH-treated group were diagnosed at a younger age (*p* = 0.031) and had lower rates of psychomotor developmental delay (*p* = 0.040) compared to those in the non-treated group. Brain pathology on imaging, etiologic classification, and the proportion of patients with cerebral palsy did not differ between treatment groups, except for 5 patients in the non-ACTH-treated group who had tuberous sclerosis (*p* < 0.001).

Most of the West syndrome cohort (110 patients, 94 %) received a variety of anti-epileptic agents over the course of the surveillance, and only 7 patients received ACTH alone. The antiepileptic medications known to affect cardiometabolic parameters were as follows: valproate in 76 patients (65 %), carbamazepine in 12 (10.3 %), and topiramate in 37 (31.6 %), with no statistically significant differences between the ACTH vs. the non-ACTH groups (59 % vs. 76.9 %, *p* = 0.057, 11.5 % vs. 7.7 %, *p* = 0.524, 30.8 % vs. 33.3 %, *p* = 0.785, respectively). Ketogenic diets were followed by 12 of the 91 Israeli patients (13.2 %): they included 11 of the 65 ACTH-treated patients (16.9 %) and 1 of the 26 non-ACTH treated patients (3.8 %) (*p* = 0.097). These data were not available in the medical files of the non-residents.

### Cardiometabolic Outcome Measures

**Fig. 2:**
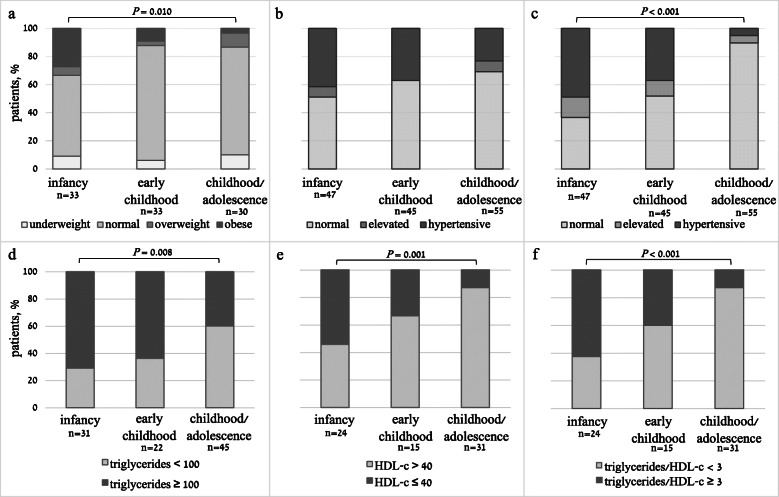
Stacked column charts of the categorical representation of weight status (a), systolic (b) and diastolic (c) blood pressure, TG levels (d), HDL-c levels (e) and triglyceride to HDL-c ratios in patients with West syndrome during infancy, early childhood, and childhood/adolescence. The proportion of patients with cardiometabolic derangements was higher in infancy compared to childhood/adolescence (obesity *P* = 0.010, diastolic hypertension *P* < 0.001, hypertriglyceridemia *P* = 0.008, low HDL-c levels *P* = 0.001, elevated TG/HDL-c ratios *P* < 0.001). Abbreviations: *TG* triglycerides; *HDL-c* high-density lipoprotein cholesterol

Data on anthropometric measurements and laboratory evaluation of the West syndrome cohort stratified according to treatment group are presented in Table 3. The mean age at last visit was 8.4 ± 5.9 years, with a median follow-up duration of 7.2 years (IQR 3.1–12.8). The mean weight and length z-scores during infancy were slightly below average (-0.58 ± 1.57, *p* = 0.010 and − 0.37 ± 1.10, *p* = 0.060, respectively), while the mean weight-to-length z-score was within average (0.29 ± 1.78, *p* = 0.384). The mean weight z-score during early childhood was slightly below average (-0.51 ± 1.49, *p* = 0.017), and the mean height and BMI z-scores were within average (-0.05 ± 1.32, *p* = 0.810 and − 0.15 ± 1.14, *p* = 0.452, respectively). The mean weight, height, and BMI z-scores during childhood and adolescence were within average (0.08 ± 1.44, *p* = 0.681, -0.28 ± 1.17, *p* = 0.196, and − 0.08 ± 1.13, *p* = 0.706, respectively). Weight status categories during infancy, early childhood, and childhood/adolescence are presented in Fig. 2a. A relatively high proportion of patients were categorized as obese during infancy compared to childhood/adolescence (27.3 % vs. 3.3 %, *p* = 0.010).

**Table 3:** Longitudinal anthropometric characteristics and laboratory evaluations of the West syndrome cohort categorized according to treatment

	ACTH treated *n* = 78	non-ACTH treated *n* = 39	*P* value
**Infancy, 0–2 years**			
Age, years	1.1 ± 0.4	1.3 ± 0.3	0.106
Anthropometric characteristics	*n* = 46	*n* = 7	
Weight z-score	-0.36 ± 1.51	-2.01 ± 1.23	**0.008**
Length z-score	-0.42 ± 1.17	-0.05 ± 0.48	0.495
Weight-to-length z-score	0.70 ± 1.45	-1.97 ± 1.86	**0.001**
BP measurements	*n* = 40	*n* = 7	
Systolic BP percentile	86.5 (66.5–97.3)	98.0 (84.0–99.0)	0.283
Diastolic BP percentile	95.0 (86.0-98.3)	91.0 (55.0–96.0)	0.403
Laboratory evaluation	*n* = 58	*n* = 11	
Glucose, mg/dL	86.5 (75.0–95.0)	91.5 (78.0-99.3)	0.154
Triglycerides, mg/dL	178.1 ± 80.7	108.6 ± 40.3	**0.037**
HDL-c, mg/dL	40.7 ± 9.8	44.3 ± 14.3	0.513
Triglycerides to HDL-c ratio	4.7 ± 2.4	2.7 ± 1.0	0.086
Early childhood, 2–6 years*	74	39	
Age, years	3.5 ± 1.1	4.2 ± 1.1	0.056
Anthropometric characteristics	n = 44	n = 10	
Weight z-score	-0.58 ± 1.64	-0.21 ± 0.49	0.486
Height z-score	-0.08 ± 1.37	0.06 ± 1.22	0.807
BMI z-score	-0.17 ± 1.25	-0.07 ± 0.68	0.841
BP measurements	*n* = 35	*n* = 10	
Systolic BP percentile	82.0 (67.5–97.5)	68.0 (29.8–87.3)	0.333
Diastolic BP percentile	92.0 (66.0–99.0)	72.0 (52.0-91.5)	0.333
Laboratory evaluation	*n* = 37	*n* = 9	
Glucose, mg/dL	85.3 (80.3–94.5)	89.0 (83.0-108.5)	0.266
Triglycerides, mg/dL	138.6 ± 61.0	78.4 ± 34.6	**0.050**
HDL-c, mg/dL	42.0 ± 11.1	44.7 ± 16.5	0.735
Triglycerides to HDL-c ratio	3.9 ± 2.7	2.6 ± 2.1	0.455
Childhood and adolescence, 6–18 years*	*n* = 53	*n* = 35	
Age, years	11.1 ± 3.4	11.9 ± 3.9	0.410
Anthropometric characteristics	n = 45	n = 15	
Weight z-score	0.27 ± 1.43	-0.51 ± 1.37	0.070
Height z-score	-0.10 ± 1.03	-1.03 ± 1.50	0.082
BMI z-score	0.05 ± 0.98	-0.58 ± 1.60	0.227
BP measurements	n = 40	n = 15	
Systolic BP percentile	77.5 (56.8–91.5)	65.0 (34.0-98.5)	0.999
Diastolic BP percentile	58.0 (37.5–79.0)	60.0 (42.0-69.5)	0.659
Laboratory evaluation	*n* = 50	*n* = 21	
Glucose, mg/dL	90.0 (80.9–98.0)	85.0 (80.0–97.0)	0.459
Triglycerides, mg/dL	100.2 ± 52.9	89.2 ± 36.5	0.485
HDL-c, mg/dL	54.6 ± 16.1	53.5 ± 14.3	0.872
Triglycerides to HDL-c ratio	2.3 ± 2.9	1.9 ± 0.6	0.723

Data are expressed as mean ± standard deviation, median (interquartile range). *P* values are between the ACTH/non-ACTH treated groups. *Of note, 74 patients in the ACTH-treated group reached early childhood and 53 patients reached childhood/adolescence, while 35 patients in the non-ACTH treated group reached childhood/adolescence at the time of data collection. Bold indicates significant. Abbreviations: *ACTH* adrenocorticotropic hormone; *BMI* body mass index; *BP* blood pressure; *HDL-c* high-density lipoprotein cholesterol; *n* number

The median systolic and diastolic BP percentiles were above average for all age groups, with a decrease over time. The median (IQR) systolic BP percentiles were 87 (66–98) in infancy, 82 (54–97) in early childhood, and 77 (55–94) in childhood/adolescence, and they tended to be higher in infancy compared to childhood/adolescence (*p* = 0.063). The median (IQR) diastolic BP percentiles were 94 (78-98.5) in infancy, 89 (60–99) in early childhood, and 59 (39–78) in childhood/adolescence, and were significantly higher in infancy than those for childhood/adolescence (*p* < 0.001). The BP categories during infancy, early childhood, and childhood/adolescence are presented in Fig. 2b&c. The proportion of systolic and/or diastolic blood pressure levels categorized as hypertensive was 58.5 % during infancy, 48.1 % during early childhood, and 26.3 % during childhood/adolescence. Systolic BP levels categorized as hypertensive tended to be higher in infancy compared to childhood/adolescence (41.5 % vs. 23.1 %, *p* = 0.080). Diastolic BP levels categorized as hypertensive were higher in infancy compared to childhood/adolescence (48.8 % vs. 5.1 %, *p* < 0.001).

The mean TG levels and TG/HDL-c ratios during infancy were elevated (162.4 ± 78.7 mg/dL and 4.3 ± 2.3, respectively). The mean TG/HDL-c were also elevated during early childhood and childhood/adolescence (3.6 ± 2.6 and 2.2 ± 2.6, respectively). The mean TG levels and TG/HDL-c ratios were higher, while the mean HDL-c levels were lower in infancy compared to childhood/adolescence (*p* < 0.001, *p* = 0.003, and *p* = 0.001, respectively). Categorical TG, HDL-c and TG:HDL-c levels during infancy, early childhood, and childhood/adolescence are presented in Fig. 2d-f. The percentage of patients with dyslipidemia was higher in infancy compared to childhood/adolescence (hypertriglyceridemia 71 % vs. 40 %, *p* = 0.008; low HDL-c 54.2 % vs. 12.9 %, *p* = 0.001; elevated TG/HDL-c ratios 62.5 % vs. 12.9 %, *p* < 0.001). The TSH and fT4 levels were within the normal range during infancy, early childhood, and childhood/adolescence, with no statistical difference between treatment groups.

During infancy, the ACTH-treated group had mean normal weight status parameters (weight and weight-to-length z-scores) which were significantly higher than those of the non-ACTH treated group (*p* = 0.008 and *p* = 0.001, respectively). In addition, they had significantly higher TG levels compared to the non-ACTH-treated group during both infancy and early childhood (*p* = 0.037 and *p* = 0.050, respectively), with no significant differences between groups during childhood and adolescence. Univariate logistic analysis revealed that ACTH-treated patients had a significantly higher risk of increased BMI z-scores (OR 1.71, 95 % CI 1.13–2.58, *p* = 0.011), increased diastolic BP percentiles (OR for 10-percentile increases 1.18, 95 % CI 1.01–1.39, *p* = 0.040) and increased TG (OR for each 10 mg/dl increase in TG 1.14, 95 % CI 1.04–1.26, *p* = 0.008), with no increased risk for elevated systolic BP percentiles, low HDL-c levels and elevated TG:HDL-c ratios.

## Discussion

The cardiometabolic characteristics of patients with West syndrome from infancy through early childhood to childhood/adolescence have not been addressed. In this observational study, patients with West syndrome exhibited increased rates of obesity, elevated BP, and dyslipidemia (hypertriglyceridemia and elevated TG:HDL-c ratios). Infants with West syndrome who were treated with ACTH were characterized by higher weight and weight-to-length values, albeit within the normal range, and higher TG levels compared to infants with West syndrome who did not receive ACTH. These differences in metabolic characteristics between treatment groups became less evident during early childhood and childhood/adolescence.

Although the proportion of preterm births among our entire cohort was high relative to normal values, most of the pregnancies were uncomplicated and had followed spontaneous conception in healthy mothers, leading to appropriate birth weights and suggesting that the in-utero environment was non-contributory. It would appear, therefore, that the weight gain during infancy and childhood was most likely related to exposure to postnatal factors. One-quarter of our study infants with West syndrome were categorized as being obese. Moreover, infants who received ACTH treatment weighed more and had normal adjusted weight-to-length ratios compared to those who were not treated with ACTH and were underweight. The lower weight status in the non-ACTH treated group could stem from their medical complexity leading to poor oral intake. However, the finding that weight status during early childhood and during childhood and adolescence did not differ between the groups suggests that the ACTH treatment contributed to the weight gain observed in the ACTH-treated group during infancy, which masks their lower weight status. Weight gain in infants with West syndrome during the course of ACTH treatment which resolved following cessation of therapy has been reported in several studies [22, 23]. In contrast, weight gain was observed after the cessation of ACTH treatment in our study cohort. Preterm birth was more frequent in the children who did not receive ACTH treatment, but prematurity is a less plausible explanation for our finding since anthropometric measurements were adjusted for gestational age. Moreover, the differences in weight cannot be attributed to the underlying etiologies since the distribution was similar between treatment groups. Thyroid function was within the normal range, thereby not supporting it having any role in weight gain.

Hypertension (HTN) was observed in the majority of infants with West syndrome. Others have reported a link between preterm birth and elevated BP levels that had been detected as early as infancy [24, 25]. It is plausible that prematurity may contribute to the elevated BP levels observed in the current work, but it could not be the sole explanatory factor since only ~ 30 % of the children were born preterm. Notably, although the vital signs were measured by registered nurses who specialize in pediatrics, it is possible that some of the measurements were spuriously elevated due to suboptimal conditions (crying toddlers, emergency room setting). ACTH therapy for the indication of infantile spasms has been reported to cause HTN [23, 26], but it could not be the sole cause of elevated BP observed in our study since BP levels were similar between the treatment groups. HTN was less evident in childhood and adolescence during which there was a normal distribution of diastolic BP values. BP was not monitored in all of the studied children, which may have led us to underestimate the incidence of HTN among children with West syndrome who had and had not been treated with ACTH. Despite significant concerns about side effects of ACTH therapy, including HTN and cardiomyopathy, there are no evidence-based guidelines to assist in the monitoring and management of these adverse effects.

Many of the patients in the current study had evidence of dyslipidemia; hypertriglyceridemia during infancy, as well as a high TG:HDL-c ratio from infancy through childhood/adolescence, although the dyslipidemia became less prominent over time. There is convincing evidence to suggest that the origins of atherosclerosis begin in childhood and that lipid abnormalities contribute to this process [19, 27]. A high TG:HDL-c ratio is associated with arterial stiffness in children and adolescents [13], and it indicates an atherogenic lipid profile and predicts the risk for development of extensive coronary disease in adults [28, 29]. Patients with epilepsy may exhibit dysregulation of thyroid functions which may impact serum lipid levels [30], however, thyroid functions were within the normal range and were unlikely to cause dyslipidemia among our study patients. Despite the efficacy of therapies, such as ACTH, many children with West syndrome will relapse and can develop other seizure types. As such, neurologists may consider selection of other anti-seizure medications and exploration of dietary interventions. Treatments that may be associated with hypertriglyceridemia include antiepileptic drugs (e.g., valproic acid, carbamazepine), and the ketogenic diet [31–33], and carbamazepine and other antiepileptic drugs have been associated with high HDL-c levels as well [31, 33]. Treatment of tuberous sclerosis complex with everolimus or sirolimus is a potential cause of dyslipidemia [34], however, none of the patients in the current study were treated with these drugs. Prematurity may also be associated with dyslipidemia later in life, but probably not with changes in triglyceride or HDL-c levels [24]. One study did find high TG levels in adolescent boys who were born preterm [35]. These explanations could account for some of our findings at best. TG levels were higher in infants and children treated with ACTH, compared to those who were not. Taken together, these findings suggest a possible link between ACTH treatment and hypertriglyceridemia.

The major limitations of this study are its retrospective nature and the relatively small sample size. Some of the data extracted from the medical files relied on the parent-reported information, such as perinatal history, and physicians documented, such as dosage and duration of the administered treatment. Yet, access to the patient’s comprehensive HMO medical files contributed to the clinical and laboratory data, medications prescribed, and thus limits information bias. Moreover, growth parameters and cardiometabolic assessment were not available for the entire cohort, introducing ascertainment bias. Notably, more data were missing for the non-resident study participants who comprised the larger proportion of the group that had not received ACTH treatment. In addition, BP measurements were obtained in several different care settings without standardized methods.

 Despite these limitations, our tertiary care center serves all sectors of the Israeli population including varied ethnic origins and socio-economic position, therefore this study should be representative of the Israeli population, apart from an under-representation of patients from the Arab sector.

In conclusion, our findings suggest that children with West syndrome have an increased prevalence of acute and subacute cardiometabolic derangements (obesity, HTN, and dyslipidemia), which are more pronounced during infancy and in ACTH-treated patients. An optimistic interpretation of our findings may be the transitory nature of the metabolic derangements. Still, they highlight the need to monitor these children at risk for cardiometabolic derangements, given that the origin of atherosclerosis begins in childhood and adolescence. The health implications in adulthood of cardiometabolic derangements during critical periods of growth and development have yet to be elucidated and warrant further investigation.

## Data Availability

The data used to support the findings of this study are available from the corresponding author upon request.

## Declarations

**Ethics Approval**

The study was approved by the Ethics Committee of the Tel-Aviv Sourasky Medical Center according to the Helsinki Declaration. Informed consent by the participants was waived by the Ethics Committee of the Tel-Aviv Sourasky Medical Center since the data were retrieved from the subjects’ medical records and all personal identification was omitted. The data were handled in accordance with the principles of Good Clinical Practice.
